# Gateway to Global Aging Data: Enabling Comparative Research on Aging, Health, and Environment

**DOI:** 10.1002/alz70860_101430

**Published:** 2025-12-23

**Authors:** Drystan Phillips

**Affiliations:** ^1^ USC, Los Angeles, CA, USA

## Abstract

**Background:**

The Gateway to Global Aging Data (Gateway) is an open research platform supporting the Health and Retirement Study International Network of Studies (HRS‐INS), a global network comprising longitudinal and multidisciplinary surveys of older populations across 46 countries. The Gateway integrates survey metadata, harmonized data files, and advanced tools to facilitate comparative studies on aging, brain health, and risk factors. Three initiatives—the Data Enclave, the Policy Explorer, and the Environmental Exposome —further enhance the platform's capacity to study complex research questions.

**Method:**

The Gateway delivers harmonized datasets and comprehensive survey metadata to enable cross‐national and longitudinal analyses of critical domains such as health, economic status, and social networks. The Data Enclave provides a secure, remote‐access computing environment for analyzing survey data while maintaining robust privacy protections and compliance with data‐sharing policies. In parallel, the Policy Explorer links individual‐level survey data to historical and current policy variables, empowering researchers to explore how macro‐level policy environments influence aging outcomes, and the Environmental Exposome integrates data on physical, natural, and chemical environments.

**Result:**

The Gateway have facilitated significant advancements in aging research. Harmonized data have revealed cross‐national differences in aging experiences. The Data Enclave has enabled secure analyses of survey data, including cognitive health trends, while the Policy Explorer has supported research into the effects of retirement policies and social support systems on individual outcomes. The Environmental Exposome stimulate research on the effects of environmental factors on health and wellbeing.

**Conclusion:**

The Gateway to Global Aging Data serves as a critical resource for advancing cross‐national research on aging. Through the integration of harmonized data and innovative tools, the Gateway empowers researchers to uncover policy‐relevant insights, addressing global aging challenges and informing strategies to promote healthy and equitable aging worldwide.

